# Nano Zinc Oxide Improves Growth Rate, Carcass Traits, Meat Chemical Composition, Serum and Tissue Mineral Profiles, Mineral Retention, and Intestinal Morphology in Broiler Chickens Compared to Inorganic and Organic Zinc

**DOI:** 10.1007/s12011-025-04716-9

**Published:** 2025-07-01

**Authors:** Hamada S. Saber, Heba A. Alian

**Affiliations:** 1 Animal Production, Agri. Faculty, Suez Canal University, Ismailia, Egypt; 2 Nutrition and Clinical Nutrition, Vet. Med. Faculty, Suez Canal University (SCU), Ismailia, 41522 Egypt

**Keywords:** Broiler, Carcass cut cup, Meat composition, Mineral profile, Nano zinc, Zinc retention

## Abstract

This study aimed to compare the impact of inorganic, organic, and nano zinc sources on growth rate, carcass traits, meat composition, mineral profiles and retention, and intestinal morphology of the broilers. A 1-day “Cobb” broiler chick (*n* = 192) was randomly distributed into six groups with four replicates. The groups were set up as follows: G1: G3 received a basal diet with 100 mg Zn/kg from inorganic zinc oxide, inorganic zinc sulfate monohydrate, and organic zinc methionine. Groups G4: G6 received a basal diet supplemented with nano zinc oxide at 20, 10, and 5 mg Zn/kg diet, respectively. The results revealed that the nano zinc oxide (NZnO) in G6 significantly increased the growth rate and improved carcass quantity and quality, as proved by a reduction in abdominal fat percentage. It was observed that nano zinc oxide achieved the highest crude protein and the lowest crude fat percentages in meat. The highest significant calcium (Ca), phosphorus (P), and zinc deposition in serum and breast, and thigh were observed in G5 and G6. Interestingly, a 20 mg NZnO/kg diet considerably raised Ca and P content in the tibia while having no significant difference in G2 or G3. A significant elevation in zinc (Zn) retention (%) was recorded in G6, followed by G4 and G5. The intestinal absorption surface area (ASA) in G5 and G6 showed a significant maximum value. The current study indicated that the lower levels of NZnO (10 and 5 mg/kg diet) showed promising results with no detrimental effect on health status, so it can safely be used in mineral premixes of broilers.

**Graphical Abstract:**

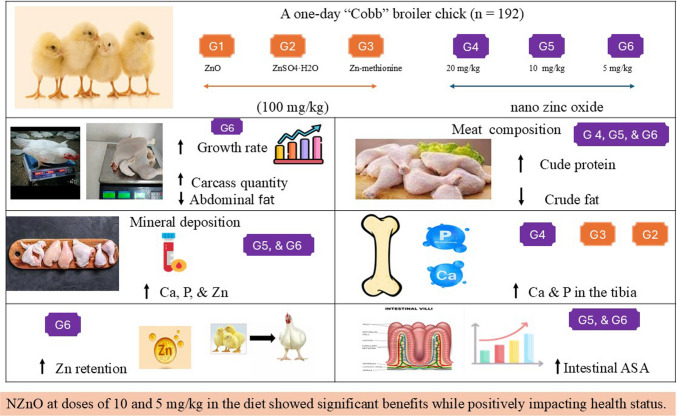

## Introduction

Poultry production is a major economic industry and the fastest-growing sector in the agriculture field because of its rapid outcome. It holds priority in meeting the population’s needs for eggs and meat, as well as processed foods. Recently, the idea of “functional foods” has attracted many investigators by enhancing poultry flesh with a variety of nutrients [58]. In producing zinc-enriched products, zinc is a vital trace mineral required for proper development and physiological functions in the body, such as immunity, antioxidant responses, and enzyme activation. Dietary zinc may impact bone growth by directly influencing protein synthesis through hormonal growth regulators, which have a positive influence on bone formation [17]. However, the gap between the ability to enhance a product and the risk of environmental pollution is important to consider. In the past, there were limits on zinc enrichment in meat, beyond which it was wasted [38]. The conventional sources of zinc in poultry feed were inorganic, specifically zinc oxide (ZnO) and zinc sulfate (ZnSO_4_). These sources exhibit inadequate bioavailability, which may induce intestinal mucosal irritation or result in elevated trace mineral excretion into the surroundings. Conversely, chelated minerals (like organic zinc) may withstand the effects of anti-nutritional factors in the digestive system and directly access the mucosal villi, leading to improved absorption [10]. However, organic zinc has a higher bioavailability than inorganic zinc,its incorporation into meals is restrained by its higher price [5].

Recently, zinc has been synthesized in nanoparticle form to reduce the pollution caused by zinc. The nanoparticle source of zinc is expected to decrease the supplemental level of zinc in the feed accordingly, which will result in a substantially lower amount of zinc escaping within the waste. Nano zinc demonstrates greater efficacy than larger zinc particles when used in smaller amounts [30]. The nanoparticles of minerals exhibit greater availability to cells due to their increased surface area, enhanced adsorption capacity, strong catalytic function, and improved surface activity [83]. Nanoparticles are currently being used to satisfy the mineral needs of livestock, thereby boosting feed efficiency and accelerating growth rates. Also, they can create pollution-free animal products and diminish environmental impacts [1]. The ability of NZnO to permeate can aid in reducing negative intestinal responses and enhance the uptake of nutrients within the digestive system [66]. Additionally, adding NZnO effectively improved body weight and feed utilization, with no negative impact on broilers’ carcass quality, blood levels, or meat quality [36]. Furthermore, a comparative study of inorganic, organic, and nano zinc-supplemented diets in broilers was conducted to assess their impact on carcass traits, meat chemical composition, serum and tissue mineral profiles, mineral retention, and intestinal morphology.

## Materials and Methods

### Management Procedure and Experimental Diets

**Fig. 1:**
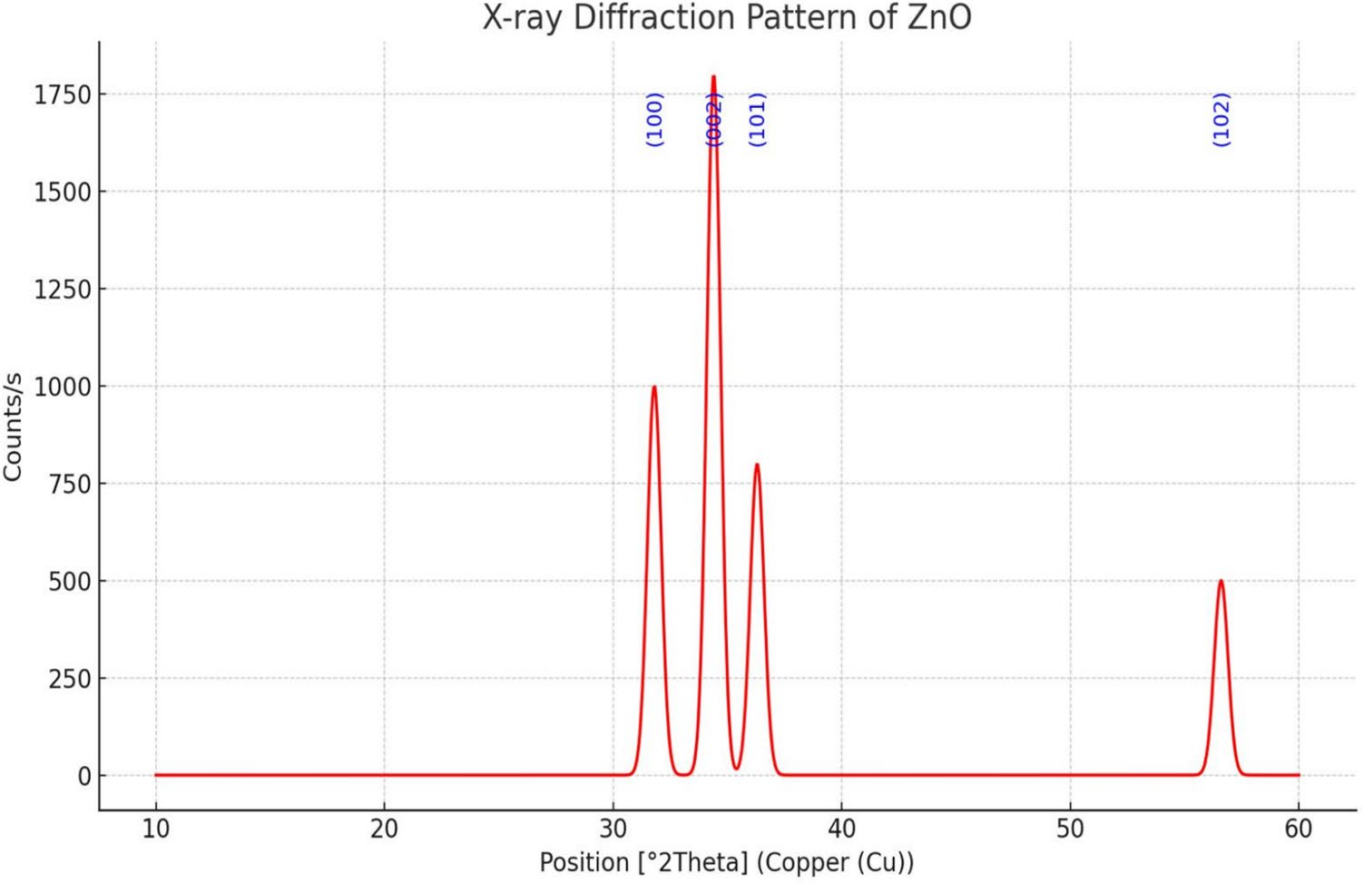
XRD pattern of nano zinc oxide (NZnO)

**Fig. 2:**
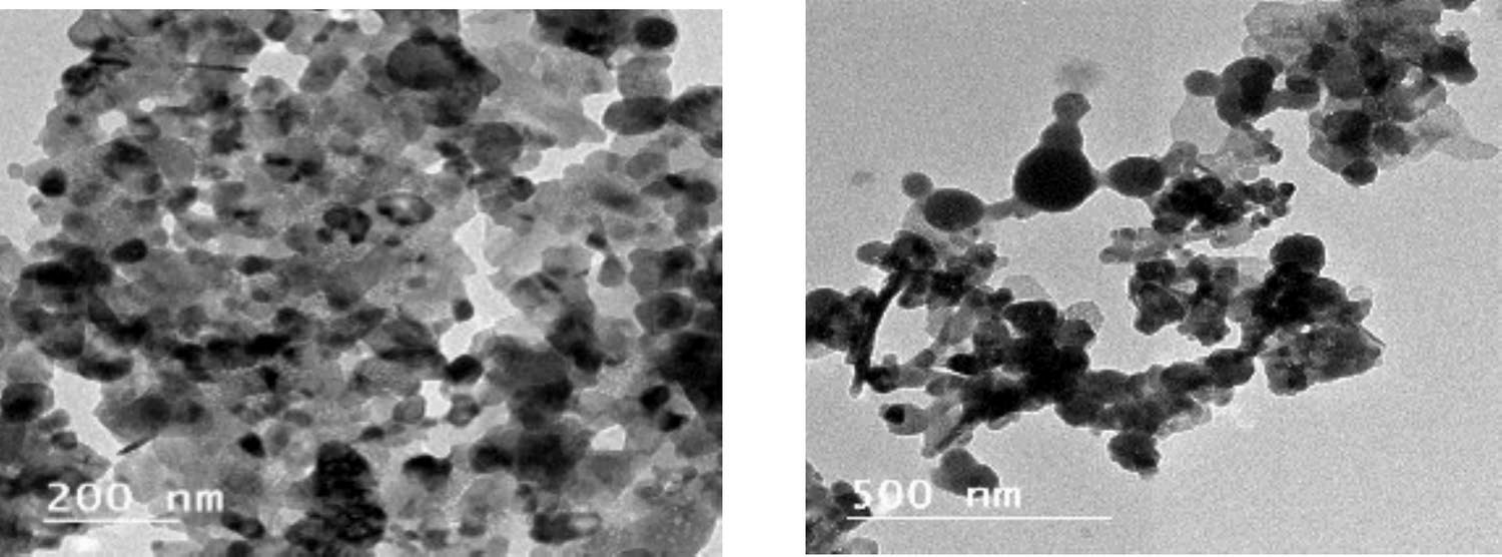
The TEM image of nano zinc oxide (NZnO)

 In our previously published online preprint, we outlined the management procedure and feeding protocol [67]. A total of 192 unsexed “Cobb” broiler chicks, 1 day old, were obtained from a commercial hatchery and subsequently divided into six groups, each consisting of four replicates with eight chicks. Each bird was provided with a diet consisting of corn and soybean, formulated to meet the nutritional requirements of chicks according to NRC [54] guidelines. The groups were defined as follows: (G1: G3) The basal diet included 100 mg Zn/kg diet from inorganic zinc oxide, inorganic zinc sulfate monohydrate, and organic zinc methionine, respectively. Groups G4: G6 received the basal diet with nano zinc oxide at levels of 20, 10, and 5 mg Zn/kg diet, respectively. The synthesis of nano zinc oxide is done according to the method declared by [42]. The X-ray diffractometer and transmission electron microscopy (TEM) are utilized to characterize the nanoparticle. Figure 1 illustrated that the diffraction data correlated closely with ICDD files, while TEM images revealed nanoparticles averaging 34 nm in size (Fig. 2). The chemical analysis of the basal diets used in the experiment was performed following AOAC [8], and the results are presented in Table 1. A three-period feeding plan was used: a period I diet (0–8 days), a period II diet (9–18 days), and a period III diet (19–35 days).

**Table 1:** The ingredients and composition of basal diets fed to broiler chicks during the rearing period (35 d)

Ingredients (g/kg)	Period I (0–8 d)	Period II (9–18 d)	Period III (19–35d)
Yellow maize	570.00	605.00	649.00
Soybean meal	300.00	270.00	243.10
Corn gluten meal (60% CP)	67.00	50.00	30.00
Vegetable oil	18.20	30.10	39.20
Calcium carbonate	12.40	10.70	10.00
Di calcium phosphate	16.80	15.70	14.00
Mineral premix^1^	2.50	2.50	2.50
Vitamin premix^2^	2.50	3.00	2.50
Sodium chloride	4.00	6.00	3.70
DL-Methionine	2.30	2.10	2.80
L-Lysine	3.30	2.90	2.20
Choline chloride	1.00	2.00	1.00
Total	1000	1000	1000
Determined and calculated value			
Metabolized energy (Kcal/kg)	3001	3101	3200
Crude protein (g/kg)*	218.20	197.10	181.6
Calcium (g/kg)	9.39	8.42	7.71
Non-phytate phosphorus (g/kg)	4.50	4.42	3.83
Zinc (mg/kg)*	26.15	24.91	23.79

^1^The 1 kg of vitamin premix: 10 × 10^6^ IU vit. A, 5 × 10^6^ IU vit. D3, 8 × 10^4^ mg vit. E, 3 × 10^3^ mg vit. K3, 3 × 10^3^ mg vit. B1, 9 × 10^3^ mg vit. B2, 4 × 10^3^ mg vit. B6, 20 mg vit. B12, 15 × 10^3^ mg pantothenic acid, 6 × 10^4^ mg nicotinic acid, 2 × 10^3^ mg folic acid and 150 mg biotin. ^2^The 1 kg of mineral premix: 5 × 10^5^ mg choline chloride, 15 × 10^4^ mg Cu, 1 × 10^3^ mg I; 4 × 10^4^ mg Fe, 1 × 10^5^ mg Mn, and 350 mg Se

^*^Determined value

During the rearing period, the chicks had unlimited access to water and feed, which was provided in a mash form. The birds were kept in battery cages and maintained in a clean environment. The chicks were reared in a temperature-controlled room where the lighting program, temperature, and relative humidity followed the procedure summarized in the Cobb500 Management Catalogue [19]. Each chick receives vaccinations according to the standard preventive schedule, which includes vaccinations for Newcastle and infectious bronchitis on the 7th and 21 st days and for infectious bursal disease on the 14th day of rearing.

### Studied Parameters

#### Growth Rate Curve

**Fig. 3:**
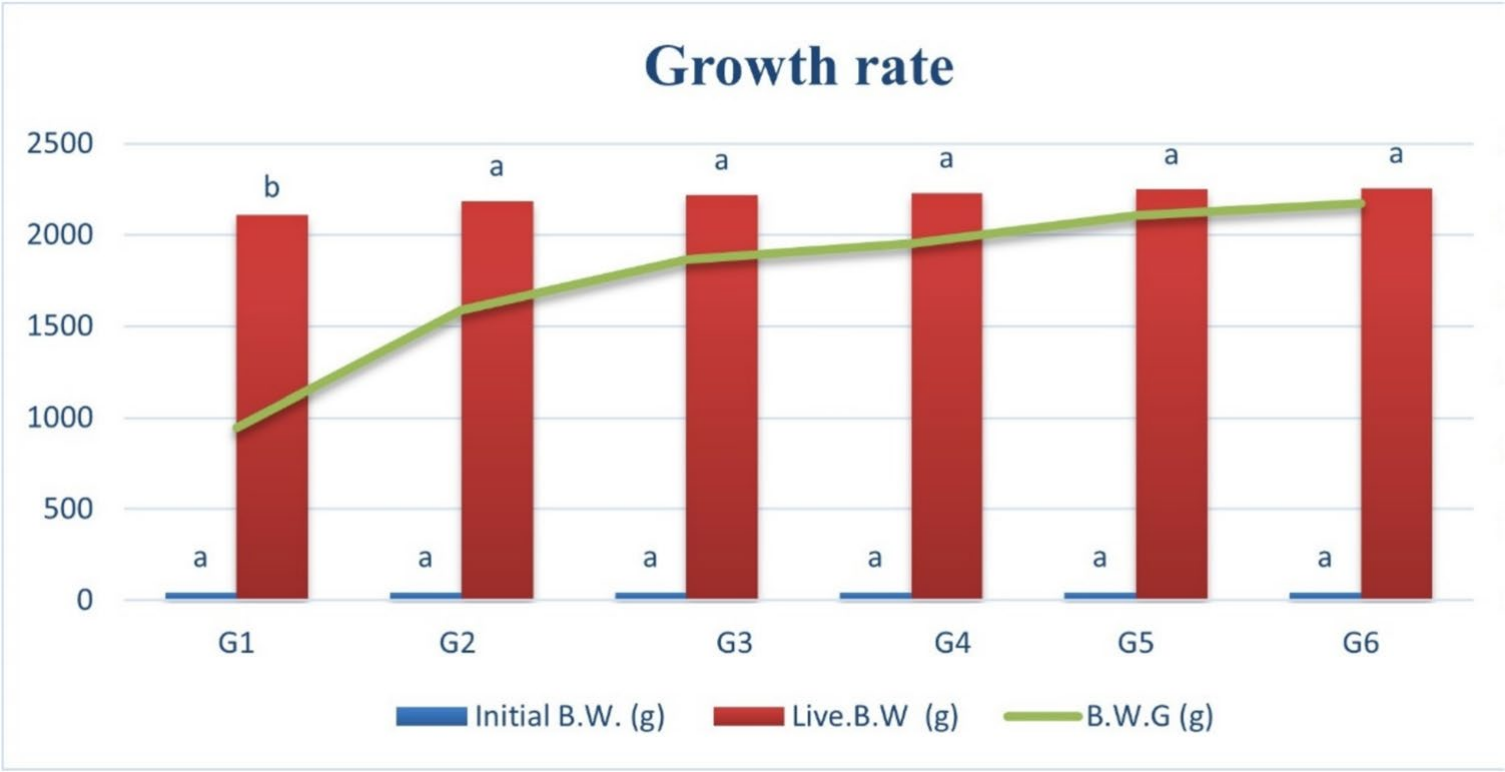
Impact of different zinc sources on the growth rate of broiler chickens at the end of the experimental period

 A total of Cobb broiler chicks were utilized in this study, exhibiting an average initial body weight of 44.10 g. Live body weight (L.B.W) and weight gain (B.W.G) were determined weekly for each experimental unit (pen). The collected data were statistically analyzed to draw the growth rate curve (Fig. 3).

#### Carcass Traits and Internal Organs’ Weight and Index Percentage

 On the 35th day, two birds were taken from each replicate and terminated by slaughter. Birds were blistered in hot water at 50–55 °C for 40 s and then plucked and eviscerated. The weights and index percentages of blood, feathers, carcass, thigh muscle, and breast muscle were measured. The dressing yield was obtained by dividing dressed weight by live body weight before slaughter. The liver, heart, gizzard, abdominal fat, proventriculus, thymus, spleen, and bursa were collected and weighed individually, then expressed as a percentage of live body weight before slaughter [4].

#### Proximate Chemical Composition of Breast and Thigh Meat

 The proximate composition of the dried meat samples (breast and thigh) was determined according to AOAC (2002). Moisture content was determined by drying in an oven at 110 °C. Crude protein was measured using the Kjeldahl method. Crude fat was extracted using petroleum ether through Soxhlet extraction. Crude ash was measured by ashing in a muffle furnace at 550 °C. Nitrogen-free extract was calculated as the difference.

#### Serum and Tissue Mineral Profiles

 The blood samples were obtained and placed into sterile tubes during the slaughtering process without any anticoagulant added. The serum samples were spun in a centrifuge at 4 °C at 5000 rpm for 10 min and subsequently stored at − 20 °C. The calorimetric determination of calcium, phosphorus, and zinc concentrations was done with the use of atomic absorption spectrometry using ready kits supplied by the Egyptian Co. for Biotechnology [84]. Mineral content in thigh and breast meat muscle was measured with the use of atomic absorption spectrometry [31]. The tibia bones were weighed after being cleansed of all adhered soft tissues. To examine bone mineralization, tibia bones were defatted for 18 h using a Soxhlet apparatus, dried overnight at 100 °C, and then ashed at 650 °C for 6 h. Total ash, phosphorus, calcium, and zinc levels in the tibia were measured [72].

#### Mineral Retention Percentage

 A digestion trial involving birds at 5 weeks of age was carried out to establish the apparent total retention of nitrogen, calcium, phosphorus, and zinc. The experiment had one bird per replicate, with four replicate birds assigned to each treatment group fed a finisher diet for six days. A 3-day adaptation period was allowed in the trial, followed by 3 days for feces collection. Birds were kept in individually designed cages with trays covered by aluminum foil for collecting excreta. Feed intake and excreta voidance were recorded daily. The collected excreta were cleared of feathers, weighed, dehydrated for 24 h at 70 °C, ground into a fine powder, and then preserved in glass jars for subsequent chemical analysis. The levels of calcium, phosphorus, and zinc were analyzed in both diets and excreta following the methods outlined by Fodor et al. [31], whereas nitrogen levels were measured using AOAC (2002) procedures.

#### Histopathological Study

 Intestine specimens from slaughtered birds were collected and immediately preserved in a 10% formalin solution for 1 day (pH = 7.2), dehydrated in ethanol (70 to 100%), cleared in xylene, and subsequently coated with paraffin. The longitudinal and transverse parts, each with a width of 5 µm, were then typically dyed with eosin stain [77]. The tissue sections were examined with a light microscope equipped with image analysis software and a full HD microscopic camera (Leica Microsystem, Germany). The intestinal parameters (villus length (VL), villous width (VW), absorption surface area (ASA), and crypt depth (CD)) were measured using image analysis software for statistical evaluation.

### Statistical Assessment

 The data were assessed via one-way ANOVA software (SPSS, version 16, USA). Duncan’s multiple-range test [22] was utilized to identify significant variations among the means at a significance level of *P* < 0.05.

## Results

### Growth Data Curve of Broiler Chicks

There are no statistically significant differences among the groups for the initial body weight. This suggests that the chicks were comparable at the beginning of the study, ensuring that any differences observed later are likely due to treatment effects. The nano zinc oxide supplemented groups, organic zinc methionine, and zinc sulphate increased the L.B.W and B.W.G significantly (*P* < 0.05) compared to the inorganic zinc oxide group (G1). However, there was a numerical increase in L.B.W and B.W.G in nano zinc groups (G4 up to G6) compared to other groups (Fig. 3).

### Carcass Traits of Broiler Chicks

The effect of different zinc sources on carcass traits is shown in Table 2. Nano zinc oxide supplemented groups and organic zinc methionine increased the body weight before slaughter, BW after bleeding, and BW after feathering either significantly (*P* < 0.05) compared to the inorganic zinc oxide group (G1) or numerically compared to chicks of G2. Nano zinc oxide at the level of 5 mg Zn/kg diet significantly increased (*P* < 0.05) the carcass weight compared to chicks of G1, G2, and G3. Also, nano zinc oxide at the levels of 20 and 10 mg Zn/kg diet significantly achieved the highest carcass weight compared to inorganic zinc sources in G1 and G2 (*P* < 0.05). In addition, nano zinc oxide, whatever the concentration, significantly (*P* < 0.05) had the highest carcass % and dressed weight and % compared to G1 and G2. Organic zinc methionine at the level of 100 mg Zn/kg diet significantly gave the greatest carcass and dressed weight compared to inorganic zinc oxide (G1) at the same level of supplementation.

**Table 2:** Carcass traits of broiler chicks at the end of the rearing period (35 d)

Group						
Parameter	G1	G2	G3	G4	G5	G6
BW before slaughter	2107.87^b^ ± 13.72	2184.37^a^ ± 32.82	2218.00^a^ ± 20.84	2228.37^a^ ± 13.34	2247.50^a^ ± 33.86	2254.50^a^ ± 9.56
BW after bleeding	2065.50^b^ ± 12.90	2142.37^a^ ± 32.14	2175.50^a^ ± 20.71	2185.37^a^ ± 12.83	2201.62^a^ ± 33.51	2206.25^a^ ± 9.33
Blood (g)	42.37^a^ ± 1.90	42.00^a^ ± 1.95	42.50^a^ ± 1.74	43.00^a^ ± 3.39	45.87^a^ ± 2.93	48.25^a^ ± 1.73
Blood (%)	2.01^a^ ± 0.08	1.92^a^ ± 0.08	1.91^a^ ± 0.07	1.93^a^ ± 0.14	2.04^a^ ± 0.13	2.14^a^ ± 0.07
BW After feathering	1961.12^b^ ± 15.30	2033.62^a^ ± 30.78	2064.25^a^ ± 19.01	2073.62^a^ ± 14.46	2086.25^a^ ± 31.47	2090.62^a^ ± 9.38
Feather (g)	104.37^b^ ± 3.41	108.75^ab^ ± 4.73	111.25^ab^ ± 3.30	111.75^ab^ ± 2.44	115.37^a^ ± 3.10	115.62^a^ ± 2.12
Feather (%)	4.95^a^ ± 0.18	4.97^a^ ± 0.20	5.01^a^ ± 0.13	5.02^a^ ± 0.13	5.13^a^ ± 0.10	5.12^a^ ± 0.09
Carcass (g)	1499.50^d^ ± 8.90	1556.25^c^ ± 20.25	1597.25^bc^ ± 16.19	1615.87^ab^ ± 9.72	1640.87^ab^ ± 19.99	1647.75^a^ ± 11.50
Carcass (%)	71.14^b^ ± 0.25	71.26^b^ ± 0.29	72.01^ab^ ± 0.45	72.51^a^ ± 0.28	73.03^a^ ± 0.34	73.09^a^ ± 0.48
Dressed (g)	1581.25^d^ ± 9.68	1639.12^c^ ± 20.82	1679.50^bc^ ± 16.83	1700.00^ab^ ± 9.99	1727.00^a^ ± 21.24	1735.25^a^ ± 11.77
Dressed (%)	75.01^c^ ± 0.28	75.06^c^ ± 0.24	75.72^bc^ ± 0.44	76.29^ab^ ± 0.33	76.86^a^ ± 0.38	76.97^a^ ± 0.48
Breast, wt. (g)	558.00^b^ ± 14.19	588.37^ab^ ± 11.04	606.62^a^ ± 12.21	603.00^a^ ± 15.42	627.25^a^ ± 20.57	629.50^a^ ± 12.08
Breast % of LBW	26.46^a^ ± 0.63	26.95^a^ ± 0.52	27.34^a^ ± 0.46	27.06^a^ ± 0.71	27.87^a^ ± 0.61	27.92^a^ ± 0.54
Thigh, wt. (g)	362.62^b^ ± 8.29	391.00^ab^ ± 16.00	398.87^ab^ ± 11.41	399.50^ab^ ± 14.08	415.87^a^ ± 19.85	416.50^a^ ± 15.00
Thigh % of LBW	17.20^a^ ± 0.40	17.87^a^ ± 0.53	18.00^a^ ± 0.59	17.91^a^ ± 0.56	18.45^a^ ± 0.63	18.45^a^ ± 0.59

Values are mean ± SE. Values in the same row with different superscripts are significantly different at *P* < 0.05

The weight of the blood and its percentage relative to live body weight did not show any significant differences across groups. The weight of the feathers varied slightly between the groups, with G6 (115.62 g) and G1 (104.37 g) displaying the maximum and minimal values, respectively. However, the percentage of feathers relative to LBW remained consistent across the groups (around 5%). It was also noticed that nano zinc oxide and organic zinc achieved the highest breast weight, either significantly (*P* < 0.05) compared to chicks in G1 or numerically with G2. Also, the thigh weight of birds in G5 and G6 was significantly higher (*P* < 0.05) compared to birds of G1. The percentage of breast and thigh relative to LBW did not show significant differences, but G6 had the highest values for both parameters.

### Internal Organs Weight and Index of Broiler Chicks

The effect of different zinc sources on the internal organ weights and indices of broiler chicks is presented in Table 3. The results indicate no significant differences in liver weights or percentages among groups. However, heart weights showed significant differences (*P* < 0.05) among experimental groups. The lowest heart weight was observed in G1, while the highest was recorded in G5. Heart percentages ranged from 0.677% in G1 to 0.750% in G5 without any significant differences. For the gizzard, significant differences (*P* < 0.05) were observed in both weight and percentage. The gizzard weight was lowest in G1 and highest in G6, while the percentage ranged from 1.49% to 1.68%, with G1 having the highest percentage and G6 the lowest. Giblet weights and percentages did not show significant differences across groups. Abdominal fat content showed no significant variations between groups, with weights ranging from 17.20 to 18.49 g, while the percentages showed the highest (*P* < 0.05) value of 0.853% and the lowest value of 0.762% for G1 and G6, respectively. Besides, there was no significant difference in proventriculus weights among experimental groups (G1, G2, G4, and G6). The proventriculus percentage was highest in G1 and lowest in G5.

**Table 3:** Internal organs weight and index percentage of broiler chicks at the end of the rearing period (35 d)

Group						
Parameter	G1	G2	G3	G4	G5	G6
Liver (g)	60.20^a^ ± 2.11	59.95^a^ ± 2.73	59.22^a^ ± 2.46	59.20^a^ ± 2.95	60.91^a^ ± 2.45	61.96^a^ ± 2.19
Liver (%)	2.85^a^ ± 0.10	2.74^a^ ± 0.11	2.67^a^ ± 0.10	2.65^a^ ± 0.13	2.71^a^ ± 0.09	2.74^a^ ± 0.09
Heart (g)	14.27^c^ ± 0.62	15.70^abc^ ± 0.61	15.22^bc^ ± 0.60	16.47^ab^ ± 0.27	16.80^a^ ± 0.28	16.76^a^ ± 0.30
Heart (%)	0.677^a^ ± 0.02	0.721^a^ ± 0.03	0.686^a^ ± 0.02	0.740^a^ ± 0.01	0.750^a^ ± 0.01	0.743^a^ ± 0.01
Gizzard (g)	7.03^c^ ± 0.20	7.28^c^ ± 0.30	7.71^bc^ ± 0.20	8.37^a^ ± 0.29	8.28^ab^ ± 0.31	8.88^a^ ± 0.29
Gizzard (%)	1.68^a^ ± 0.05	1.61^ab^ ± 0.06	1.60^ab^ ± 0.04	1.62^ab^ ± 0.05	1.50^b^ ± 0.05	1.49^b^ ± 0.04
Giblet (g)	81.75^a^ ± 2.48	82.87^a^ ± 2.92	82.25^a^ ± 2.52	84.12^a^ ± 2.68	86.12^a^ ± 2.16	87.50^a^ ± 2.07
Giblet (%)	3.86^a^ ± 0.11	3.79^a^ ± 0.12	3.70^a^ ± 0.10	3.77^a^ ± 0.12	3.82^a^ ± 0.08	3.88^a^ ± 0.08
Abdominal fat (g)	18.00^a^ ± 0.58	17.75^a^ ± 0.78	18.49^a^ ± 0.53	17.84^a^ ± 0.74	17.83^a^ ± 0.56	17.20^a^ ± 0.61
Abdominal fat (%)	0.853^a^ ± 0.02	0.810^ab^ ± 0.02	0.833^ab^ ± 0.02	0.801^ab^ ± 0.03	0.793^ab^ ± 0.02	0.762^b^ ± 0.02
Proventriculus (g)	8.36^ab^ ± 0.21	8.13^abc^ ± 0.20	8.58^a^ ± 0.14	8.24^ab^ ± 0.24	7.56^c^ ± 0.18	7.77^bc^ ± 0.19
Proventriculus (%)	0.395^a^ ± 0.01	0.372^ab^ ± 0.01	0.387^a^ ± 0.009	0.368^ab^ ± 0.01	0.338^b^ ± 0.01	0.343^b^ ± 0.009
Spleen (g)	1.11^bc^ ± 0.16	1.04^c^ ± 0.09	1.24^abc^ ± 0.12	1.35^abc^ ± 0.09	1.47^ab^ ± 0.09	1.50^a^ ± 0.13
Spleen (%)	0.052^ab^ ± 0.008	0.046^b^ ± 0.003	0.055^ab^ ± 0.004	0.058^ab^ ± 0.004	0.067^a^ ± 0.003	0.065^a^ ± 0.005
Thymus (g)	7.41^b^ ± 0.36	7.60^b^ ± 0.23	8.14^b^ ± 0.27	8.97^a^ ± 0.27	9.27^a^ ± 0.30	9.53^a^ ± 0.27
Thymus (%)	0.351^c^ ± 0.01	0.348^c^ ± 0.009	0.367^bc^ ± 0.01	0.402^ab^ ± 0.01	0.413^a^ ± 0.01	0.423^a^ ± 0.01
Bursa (g)	0.887^b^ ± 0.05	0.867^b^ ± 0.03	1.00^b^ ± 0.05	1.09^ab^ ± 0.07	1.23^a^ ± 0.10	1.27^a^ ± 0.09
Bursa (%)	0.042^c^ ± 0.003	0.040^c^ ± 0.001	0.045^bc^ ± 0.002	0.047^abc^ ± 0.003	0.055^ab^ ± 0.005	0.056^a^ ± 0.004

Values are mean ± SE. Values in the same row with different superscripts are significantly different at *P* < 0.05

Nano zinc oxide at the level of 5 mg Zn/kg diet had the best results in spleen weight compared to G1 and G2 (*P* < 0.05). Also, the lower levels of NZnO (G5 and G6) significantly (*P* < 0.05) gave the highest spleen % value compared to G2 and achieved the greatest bursa and thymus weight and % (*P* < 0.05) compared to G1, G2, and G3. A significant elevation in bursa% (*P* < 0.05) was recorded in nano zinc oxide groups (G5 and G6) than inorganic zinc groups (G1 and G2).

### Proximate Chemical Composition of Breast and Thigh Meat

The chemical composition of breast and thigh meat is stated in Table 4. It was observed that nano zinc oxide (G6) at the level of 5 mg Zn/kg diet significantly (*P* < 0.05) had the lowest moisture content compared to zinc oxide (G1). Nano zinc oxide and organic zinc significantly improved the crude protein content in the breast meat compared to G1 and G2, where the highest significant value was achieved in G5 and G6. It was also reported that nano zinc oxide groups significantly (*P* < 0.05) achieved the lowest crude fat content in the breast meat compared to organic (G3) and inorganic zinc groups (G1 and G2), and the lowest value was obtained in G5 and G6. The Ash and NFE in the meat of the breast were not significantly affected by different zinc sources.

**Table 4:** Proximate chemical composition (%) of breast and thigh meat at the end of the rearing period (35d)

Group						
Parameter	G1	G2	G3	G4	G5	G6
Breast						
Moisture	7.03^a^ ± 0.16	6.94^ab^ ± 0.15	6.89^ab^ ± 0.11	6.67^ab^ ± 0.14	6.62^ab^ ± 0.11	6.51^b^ ± 0.11
Crude protein	54.91^d^ ± 0.14	55.74^c^ ± 0.23	56.56^b^ ± 0.21	57.13^b^ ± 0.27	58.42^a^ ± 0.26	58.80^a^ ± 0.34
Crude fat	33.87^a^ ± 0.11	32.31^b^ ± 0.26	31.89^b^ ± 0.19	31.07^c^ ± 0.20	29.89^d^ ± 0.14	29.29^d^ ± 0.31
Ash	3.59^a^ ± 0.17	3.86^a^ ± 0.14	3.98^a^ ± 0.19	4.17^a^ ± 0.20	4.18^a^ ± 0.06	4.18^a^ ± 0.24
NFE	0.59^a^ ± 0.13	1.15^a^ ± 0.23	0.68^a^ ± 0.19	0.95^a^ ± 0.14	0.89^a^ ± 0.25	1.21^a^ ± 0.27
Thigh						
Moisture	6.20^a^ ± 0.10	6.09^a^ ± 0.16	6.14^a^ ± 0.13	5.90^ab^ ± 0.15	5.77^ab^ ± 0.07	5.55^b^ ± 0.19
Crude protein	52.10^e^ ± 0.30	52.43^de^ ± 0.26	53.38^cd^ ± 0.26	54.03^c^ ± 0.33	54.99^b^ ± 0.40	56.97^a^ ± 0.39
Crude fat	37.53^a^ ± 0.22	37.18^ab^ ± 0.19	36.75^b^ ± 0.16	35.66^c^ ± 0.16	34.39^d^ ± 0.17	33.63^e^ ± 0.24
Ash	2.50^a^ ± 0.11	2.37^a^ ± 0.15	2.10^a^ ± 0.14	2.07^a^ ± 0.10	2.42^a^ ± 0.22	2.37^a^ ± 0.06
NFE	1.67^b^ ± 0.19	1.93^ab^ ± 0.15	1.64^b^ ± 0.23	2.34^a^ ± 0.17	2.43^a^ ± 0.15	1.48^b^ ± 0.17

Values are mean ± SE. Values in the same row with different superscripts are significantly different at *P* < 0.05

In thigh meat, the moisture content in birds fed with nano zinc oxide at the level of 5 mg Zn/kg significantly (*P* < 0.05) had the lowest value compared to other groups. It was stated that G6, followed by G5, significantly (*P* < 0.05) achieved greater crude protein and the lowest crude fat content in thigh meat. It was also reported that G3, G4, and G5 significantly gave the highest crude protein, and the lowest crude fat content compared to G1. No significant differences were detected in the ash content of thigh meat among experimental groups. Besides, NFE was not significantly affected by dietary zinc in G1, G2, G3, and G6.

### Serum and Tissue Mineral Profiles of Broiler Chicks

The serum and tissue mineral profiles of broiler chicks are described in Table 5. In serum, the Ca and P levels were high (*P* < 0.05) in the lower levels of NZnO groups (G5 and G6) compared to G1, G2, and G3. Also, organic zinc methionine (G3) significantly (*P* < 0.05) elevated the serum Ca and P levels compared to chicks in G1 and numerically compared to G2. Also, nano zinc oxide in G5 and G6 significantly (*P* < 0.05) achieved the best results in serum Zn level compared to chicks in G1 and G2, G3, and G4. Also, the organic zinc methionine elevated the Zn level either significantly (*P* < 0.05) compared to the zinc oxide (G1) or numerically compared to zinc sulfate (G2). In breast meat muscle, nano zinc oxide in G5 and G6 significantly had (*P* < 0.05) the greatest Ca, P, and Zn values compared to chicks in G1, G2, and G3. It was also observed that zinc methionine significantly (*P* < 0.05) promoted the level of Ca, P, and Zn compared to inorganic enriched groups (G1 and G2).

**Table 5:** Serum and tissue mineral profile of broiler chicks at the end of the rearing period (35 d)

Group						
Parameter	G1	G2	G3	G4	G5	G6
Serum						
Calcium (mg/dL)	9.1^e^ ± 0.23	9.51^de^ ± 0.25	9.90^cd^ ± 0.26	10.51^bc^ ± 0.25	11.06^ab^ ± 0.28	11.29^a^ ± 0.19
Phosphorus (mg/dL)	4.72^e^ ± 0.16	5.23^de^ ± 0.26	5.81^cd^ ± 0.24	6.29^bc^ ± 0.21	6.69^ab^ ± 0.27	6.97^a^ ± 0.20
Zinc (µg/dL)	98.53^d^ ± 3.05	106.93^cd^ ± 3.41	113.94^bc^ ± 3.08	122.18^b^ ± 3.19	135.49^a^ ± 2.93	137.95^a^ ± 2.76
Breast muscle						
Calcium%	0.46^e^ ± 0.006	0.51^d^ ± 0.008	0.55^c^ ± 0.005	0.59^b^ ± 0.007	0.62^a^ ± 0.005	0.64^a^ ± 0.006
Phosphorus%	0.65^e^ ± 0.006	0.68^d^ ± 0.007	0.75^c^ ± 0.005	0.77^bc^ ± 0.01	0.78^b^ ± 0.01	0.82^a^ ± 0.01
Zinc(ppm)	210.87^f^ ± 2.31	219.87^e^ ± 1.28	228.37^d^ ± 1.72	235.12^c^ ± 1.51	241.12^b^ ± 1.27	252.87^a^ ± 2.09
Thigh muscle						
Calcium%	0.42^e^ ± 0.005	0.43^e^ ± 0.006	0.46^d^ ± 0.005	0.52^c^ ± 0.01	0.57^b^ ± 0.007	0.62^a^ ± 0.01
Phosphorus%	0.60^e^ ± 0.005	0.62^e^ ± 0.007	0.65^d^ ± 0.004	0.67^c^ ± 0.005	0.69^b^ ± 0.008	0.73^a^ ± 0.005
Zinc (ppm)	417.25^e^ ± 3.94	436.50^d^ ± 1.54	444.75^c^ ± 1.66	457.75^b^ ± 0.90	462.88^b^ ± 1.61	493.25^a^ ± 2.30
Tibia measurements						
Calcium (%)	32.31^a^ ± 0.29	32.10^ab^ ± 0.56	31.96^ab^ ± 0.48	30.98^bc^ ± 0.45	29.91^cb^ ± 0.31	29.72^d^ ± 0.31
Phosphorus (%)	13.32^a^ ± 0.25	13.02^ab^ ± 0.17	12.98^ab^ ± 0.21	12.48^bc^ ± 0.22	12.19^c^ ± 0.29	12.02^c^ ± 0.17
Zinc (ppm)	163.32^b^ ± 0.46	163.86^b^ ± 0.51	167.15^a^ ± 0.43	162.67^bc^ ± 1.22	162.21^bc^ ± 1.32	160.44^c^ ± 0.62
Tibia ash (%)	36.66^a^ ± 0.83	36.03^ab^ ± 0.53	35.86^ab^ ± 0.15	34.27^bc^ ± 0.89	33.89^bc^ ± 0.94	32.19^c^ ± 0.45

Values are mean ± SE. Values in the same row with different superscripts are significantly different at *P* < 0.05

In thigh meat muscle, in comparison with G1, G2, and G3, there was a significant (*P* < 0.05) increasing trend in Ca, P, and Zn levels when the diet was supplemented with nano zinc oxide from G5 up to G6, which had the highest level of these minerals. Also, the organic zinc methionine group (G3) gave the highest Ca, P, and Zn levels compared to G1 and G2. Regarding the results of tibia measurements, significantly (*P* < 0.05) higher Ca and P levels were observed in G1, G2, and G3 at 100 mg Zn/kg diet, whatever the sources, followed by G4 (20 mg NZnO/kg diet). The organic zinc (G3) significantly achieved (*P* < 0.05) the best results in tibia zinc level compared to G1, G2, G4, and G5, which did not differ significantly from each other. The results showed that the bone ash content in G2, G3, G4, and G5 did not show a significant effect*.*

### Mineral Retention Percentage of Broiler Chicks

The mineral retention percentage at the end of the rearing period is presented in Table 6. The dietary zinc source in different experimental groups did not show any significant effect (*P* < 0.05) on nitrogen retention in broiler chickens. The higher calcium retention percentage (*P* < 0.05) was detected in G3 and G4 compared to G1 and G6 but did not show significant differences from each other. Also, it was revealed that dietary zinc in G2 and G3 as inorganic zinc sulfate and organic zinc methionine, respectively, significantly achieved the highest P retention percentage (*P* < 0.05) in comparison to chicks in G1, G5, and G6 and numerically to G4. Nano zinc oxide in G6 at the level of 5 mg Zn/kg diet significantly achieved the best Zn retention % (*P* < 0.05) compared to chicks in G1, G2, G3, and G4 and numerically to G5. It was observed that nano zinc in G4 and G5 and the organic zinc methionine group had the maximum zinc retention percentage significantly (*P* < 0.05) compared to G1 and numerically compared to G2.

**Table 6:** Mineral retention (%)of broiler chicksat the end of the rearing period(35d)

Group						
Parameter	G1	G2	G3	G4	G5	G6
Nitrogen retention	88.10^a^ ± 0.35	89.21^a^ ± 0.42	89.34^a^ ± 0.54	88.85^a^ ± 0.48	88.27^a^ ± 0.22	88.12^a^ ± 0.20
Calcium retention	44.37^d^ ± 0.71	46.24^bcd^ ± 0.91	50.47^a^ ± 0.88	47.89^ab^ ± 0.86	47.75^abc^ ± 0.70	44.99^cd^ ± 1.20
Phosphorus retention	45.51^b^ ± 0.31	50.19^a^ ± 1.56	51.16^a^ ± 1.25	48.58^ab^ ± 0.86	46.81^b^ ± 0.71	46.35^b^ ± 0.96
Zinc retention	52.75^c^ ± 1.09	60.21^b^ ± 1.07	60.30^b^ ± 0.23	61.58^b^ ± 0.62	62.69^ab^ ± 0.77	64.80^a^ ± 0.60

Values are mean ± SE. Values in the same row with different superscripts are significantly different at *P* < 0.05

### Intestinal Morphology of Broiler Chicks

**Fig. 4:**
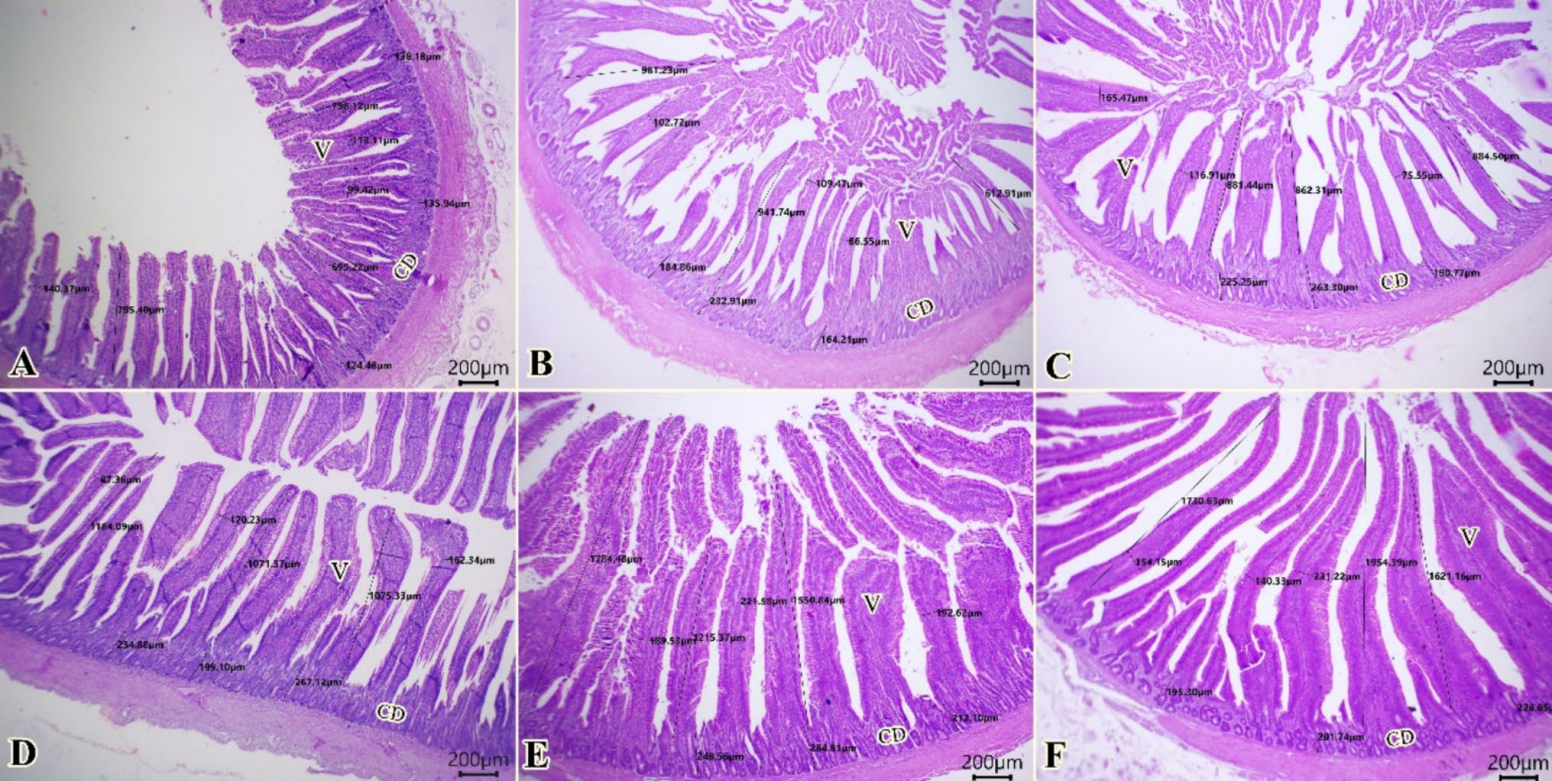
Microscopical examination of E-stained sections of intestine (scale bar 200 µm) showing improved intestinal morphometric from G1 to G6 (**A**–**F**). V “mucosal villi” and CD “crypt depth”

All tissue sections of the experimental groups showed normal morphology of mucosal villi, submucosal layer, and muscular layers. Intestinal parameters (villus length, villous width, absorption surface area, and crypt depth) gradually boosted from G1 to G6 (Fig. 4A–F). In addition, Table 7 displayed the effects of zinc sources on statistically measured intestinal parameters (VL, VW, ASA, and CD). The data showed that NZnO at the levels of 10 and 5 mg Zn/kg diet significantly (*P* < 0.05) achieved the greatest VL value compared to G1, G2, G3, and G4. Also, birds in G4 (20 mg NZnO/kg diet) had the greatest VL value significantly (*P* < 0.05) compared to G1 and numerically compared to G2 and G3. Besides, VW was elevated in G5 and G6 either significantly (*P* < 0.05) compared to G2 or numerically to G1, G2, and G4. The significantly highest absorption surface area, ASA (*P* < 0.05), was detected in nano zinc supplemented groups G5 and G6, followed by G4 among all experimental groups. Also, organic zinc methionine significantly had the best results in ASA (*P* < 0.05) in comparison to chicks in G1 and G2. On the other hand, the significantly lowest CD (*P* < 0.05) was noticed in the zinc oxide group (G1) among all experimental groups.

**Table 7:** Intestinal morphologyof broiler chicks at the end of the rearing period (35d)

Group						
Parameter	G1	G2	G3	G4	G5	G6
Villous length (VL) µm	762.91^c^ ± 33.85	845.29^bc^ ± 116.74	876.08^bc^ ± 6.94	1110.26^b^ ± 36.93	1516.89^a^ ± 165.162	1768.73^a^ ± 98.06
Villous width (VW) µm	117.63^bc^ ± 12.03	99.58^c^ ± 6.80	119.31^bc^ ± 25.98	139.97^abc^ ± 26.40	191.24^a^ ± 17.925	175.23^ab^ ± 28.27
Absorption surface area (ASA) “mm^2^”	0.0863^d^ ± 0.003	0.0787^d^ ± 0.005	0.1167^c^ ± 0.01	0.1703^b^ ± 0.008	0.2813^a^ ± 0.006	0.2923^a^ ± 0.01
Crypt depth (CD) µm	132.86^b^ ± 4.24	193.99^a^ ± 20.35	219.77^a^ ± 15.40	217.03^a^ ± 10.32	248.49^a^ ± 20.98	235.09^a^ ± 25.25

Values are mean ± SE. Values in the same row with different superscripts are significantly different at *P* < 0.05. *VL* were defined from their tip to the base. *VW* were measured at the half height point. *ASA* (mm^2^) = villus height × villus width

## Discussion

Regardless of concentration, nano zinc oxide significantly enhanced the growth rate, carcass weight, dressed weight, and percentage. There was an improvement in the breast weight of birds in nano zinc oxide groups (G4, G5, and G6). Also, an increase in the thigh weight of birds in G5 and G6 was observed. This improvement may be clarified based on the upgrade in growth performance parameters because of proper nutrient utilization efficiency. Meanwhile, zinc is essential for optimal growth and overall health. [56]. Also, it is enhancing the hormone secretion [81] as well as crucial for DNA and protein synthesis [59]. Compared to other zinc sources, the benefits of zinc were more noticeable in groups supplemented with nano zinc oxide because of its better bioavailability. The present data agree with those attained by Ebrahimnezhad et al. [23] who indicated that a diet containing 60 or 90 ppm nano ZnO per kg significantly enhanced broiler chicks’ breast and thigh weights compared to the control. The group receiving a 100 mg Zn/kg diet with nano zinc exhibited the highest carcass and dressed percentages [24]. Conversely, Akhavan-Salamat and Ghasemi [2] reported that the carcass traits, dressing, breast, or thigh percentage, were not affected by different sources (ZnO, Zn-Met, and NZnO) or levels (20, 40, and 80 mg/kg diet). Moreover, neither the carcass nor the breast weight showed any significant changes due to the dietary treatment of 100 ppm of ZnO and 25–50 ppm of NZnO [12]. The difference in the data obtained relates to the dose of nano zinc oxide used or the length of the experiment. Also, organic zinc methionine significantly improved carcass, dressed, and breast weight. This aligns with the previous research, indicating that organic Zn sources are more bioavailable than inorganic ones [21]. The current data agree with Rossi et al. [64], who found that organic Zn at 45 and 30 mg/kg diet enhanced breast weight and carcass yield, respectively. Jahanian et al. [37]showed that organic Zn at 120, 80, or 40 mg/kg feed raised the breast yield of broilers. Additionally, El-Husseiny et al. [26] clarified that the birds received a meal enhanced with 50% organic elements of Mn, Zn, and Cu, significantly increasing dressing and breast muscle percentages. Kwiecień et al. [43] reported that feeding 50 and 25 mg of Zn as glycine chelate per kg of feed enhanced the relative weights of leg muscles and breasts. However, Hess et al. [33], Zakaria et al. [86], and Qudsieh et al. [60] reported that organic Zn sources had no significant impact on carcass cut-part and yield in broilers.

There was a recorded non-significant effect of zinc sources on different organ index weights (blood, liver, and giblets) and percentages (blood, feather, breast % of LBW, thigh % of LBW, liver, heart, and giblets). The non-significant effects of zinc supplementation on digestive organs may be due to the finding of the non-significant impact of zinc on feed intake; thus, the amount of nutrients for the increase in organ weight was not changed. This coincided with Akhavan-Salamat and Ghasemi [2] who observed that dietary zinc sources (Zn oxide, Zn-Met, and NZnO) and their rates (20, 40, and 80 ppm) did not affect the weights of the heart, liver, and abdominal fat. Also, Bami et al. [12] reported that the gizzard, heart, and abdominal fat weights remained unchanged with 100 mg ZnO and 50 and 25 mg NZnO/kg. Dietary Zn treatments, including 70 ppm from ZnSO_4_, 70 ppm from Zn amino acid complex, and 30, 50, 70, and 90 ppm from NZnO, did not change the relative weight of the liver, pancreas, gizzard, and abdominal fat pad [28]. In contrast to our data, Mohammadi et al. [49] noted an improvement in the digestive organ weight of broilers who received 100 ppm of NZnO. Mahmoud et al. [47] observed that the edible yield in the 20 ppm NZnO group was significantly higher compared to the 40 ppm NZnO group. Additionally, birds receiving diets with 80 and 20 mg/kg of nano NZnO exhibited higher giblet percentages. Besides, the highest edible part percentage was observed by feeding an 80 mg Zn/kg diet as nano zinc oxide [24].

Nano zinc at a level of 5 mg Zn/kg diet also improved carcass quality, as proved by a reduction in abdominal fat percentage. Abdominal fat is not transformed into meat, so it is believed to be useless for efficient feed utilization [62]. Minimizing fat buildup in chickens is a key objective for the broiler industry. This is influenced by consumers’ need for high-quality products that meet health standards. Animal products with high fat content significantly contribute to coronary heart disorders. So, lowering abdominal fat in poultry not only reduces industry costs but also boosts feed efficiency [34]. Accordingly, NZnO supplementation in broiler diets improves carcass quality by lowering the percentage of abdominal fat [36].

Dietary levels of 10 mg and 5 mg Zn/kg diet of nano zinc oxide significantly increased the weights of the spleen, bursa, and thymus, with the 5 mg NZnO/kg diet showing the highest spleen weight. These organs are part of the defense system and can protect the bird against infectious attacks [16]. The primary lymphoid organ in poultry, the bursa of Fabricius, is essential for the differentiation of B lymphocytes [70]. While, Kidd et al. [40] indicated that the organs’ size was influenced by the zinc content in the poultry diet, decreasing when zinc levels were low. Smith and Hunt [73] reported that the spleen supported lymphocyte growth, which has a critical role in the humoral and cellular immune responses. In the same time, Møller and Erritzøe [50]proposed that there was a direct relationship between humoral immunity and spleen size; a larger spleen is associated with higher humoral immunity and vice versa. According to the current data, replacing inorganic zinc oxide with reduced levels of nano zinc results in an increase in thymus and spleen weight [27]. Additionally, Mahmoud et al. [47]found that 10 ppm of nano zinc oxide significantly increased spleen weight. Furthermore, dietary NZnO at 60 and 40 mg/kg improved the bursa of Fabricius weight [24]. On the other hand, Bartlett and Smith [13]said that the thymus, bursa, and spleen were not significantly influenced by the rate of zinc supplementation in the diet. Also, Eskandani et al. [28]implied that the weight of broiler chicken immune organs was unchanged by dietary zinc sources: ZnSO4, Zn amino acid complex, or NZnO.

In breast meat, the lowest moisture content was observed in chicks that received a diet with 5 mg of nano zinc oxide per kg. Additionally, it was observed that nano zinc oxide groups resulted in the highest percentages of crude protein and the lowest percentages of crude fat in breast meat. Organic zinc had the highest crude protein compared to inorganic zinc. Adding various zinc sources had no impact on the Ash and NFE. Nano zinc oxide (G6) significantly achieved the lowest thigh moisture content. G6, followed by G5, then G4, and G3, had the superior crude protein and the least crude fat. The ash and NFE were not affected by zinc supplementation. These were in agreement with Liu et al. [46], who found that the breast moisture content was lower in chicks fed zinc-enriched diets. Khah et al. [39] stated that 60 and 90 ppm of nano zinc oxide significantly achieved the greatest dry matter and crude protein and the lowest ether extract of breast and thigh meat. Also, Norouzi et al. [53] reported an increase in breast protein percent by zinc addition. Elevated crude protein levels could result from improved nutrient digestibility, particularly proteins, and growth rate enhancement caused by zinc supplementation. Additionally, zinc is crucial for protecting pancreatic cells from oxidative stress. It helps the pancreas for adequate digestive enzyme secretion, resulting in increased nutrient digestibility [88]. Our results are the following Ao et al. [7], who observed that varying levels of zinc in the diet did not influence the breast ash of broilers. In contrast, Tronina et al. [80] reported that the breast crude protein of broilers in the zinc-glycine group was lower than in the control and zinc oxide groups. Also, Liu et al. [46] mentioned that the thigh fat percentage was high in zinc-supplemented diets compared to the control one. The variations in studies might relate to the duration of the experimental phase or the background of zinc in the diet and rearing conditions.

Estimating the amount of minerals deposited in specific tissues, such as blood, muscle, and tibia, is the most popular technique for determining the bioavailability of minerals [76]. Also, serum Zn levels can be used to indicate the amount of zinc assimilated by birds during rearing. Thus, low serum Zn levels indicate an early stage of deficiency [34]. Also, the zinc content in breast meat muscle is boosted in broiler chicken feed Zn in a dose-dependent manner [69]. The present findings stated that NZnO supplementation improved the efficacy of Ca, P, and Zn deposition in serum and breast and thigh meat of broiler chickens. Additionally, the highest significant values are observed in lower levels of nano zinc supplementation (G5 and G6). It was observed that zinc methionine (G3) gave the highest Ca, P, and Zn levels in the serum and meat of broilers. As stated by Zakaria et al. [87], the lowest serum calcium contents were observed in birds fed inorganic zinc compared to organic Zn. Raje [61] explained that, in comparison to inorganic zinc, nano zinc elevated the serum zinc level in Wistar rats. Since zinc is an essential part of the enzymes participating in vitamin D3 synthesis, it controls the Ca and P absorption in the intestines, kidneys, and bones [6]. The better Zn deposition resulting from the NZnO source demonstrates a higher bioavailability than the traditional one. This high bioavailability of NZnO can alter the deposition of minerals by improving their reactions with biological surfaces [44]. Also, NZnO can enter hepatocytes via the interstitial spaces of the bloodstream due to its nanoparticle size [74]. Another explanation could be due to the role of metallothionein, a protein that modifies the pool and turnover of trace minerals. It is produced in the body's response to dietary Zn, capable of binding seven zinc atoms to every protein molecule [20]. A higher concentration of zinc in bodily tissues could be caused by an increase in this zinc-binding protein [79].

The measurements of the tibia were modified by mineral supplements in the feed [25]. High zinc levels in the diet tended to raise the calcium and phosphorus content in the tibia. In our study, we observed that increasing zinc levels in the diet improves the tibia Ca and P values, though the influences of inorganic and organic zinc were similar. Interestingly, a 20 mg NZnO/kg diet in G4 considerably raises Ca and P content in the tibia while having no significant difference from G2 or G3 at the same level (100 mg Zn/kg diet). These data revealed that zinc sources, whatever the source, supported Ca and P deposition in the tibia, showing no antagonistic effect. These were like previously reported results [52, 63, 75, 82].

The Tibia Zn level was observed to be significantly greater in G3 (zinc methionine) compared to G1, G2, G4, and G5, which were relatively like each other. This was agreed with Behjatian Esfahani et al. [14] who reported that Zn-Met-provided zinc was more bioavailable compared to ZnSO4 or ZnO. Bones act as a reserve for Zn within the body, and a suitable Zn level is important for bone growth and mineralization [65]. Zinc metabolism in bones is dynamic, with zinc relocating from bones to different body parts based on tissue needs. However, the explanation for the higher tibia zinc level in G1 (100 mg ZnO/kg diet) compared to G6 (5 mg NZnO/kg diet) might be due to the higher level of dietary zinc. However, G1 does not differ significantly from nano zinc oxide supplemented groups G4 and G5 at the 20 and 10 mg NZnO/kg diet, respectively. Moreover, many researchers reported that the zinc content in the tibia ash is significantly affected by consumption [11]. So, the dietary Zn level affects Tibia Zn content more than the Zn source itself. The better bioavailability of nano zinc resulted in its greater absorption, which is not only deposited in bone in higher content but also influences cell metabolism by its impacts on growth and hormonal factors to a greater extent. These conclusions are confirmed by Zhao et al. [89] who found a higher amount of zinc in the hepatocytes of chicks fed nano zinc than traditional zinc.

In contrast, Zhao et al. [90] clarified that by replacing 50% of the inorganic Zn, Cu, and Mn with organic form, there was no significant difference detected for tibia zinc content, which might be due to a short period of feeding. Sahraei et al. [68] cited that the amount of tibia Zn did not differ with the zinc addition (100, 150, or 200 ppm) as organic Zn or inorganic ZnO in broilers. The same findings were reported by Mwangi et al. [51].

The bone ash content in G2, G3, G4, and G5 did not show any differences*.* This data agrees with Sunder et al. [75]andSwiatkiewicz and Koreleski [78] who reported that dietary organic zinc had no significant influence on the relative weight of the tibia and ash content of the tibia and toe of the laying birds.

It is noticeable that broilers presented positive N, Ca, P, and Zn retention values, as mineral retention was calculated as the difference between mineral intake and mineral excretion. The data obtained revealed that the different zinc sources showed no significant impact on the N retention percentage. The higher calcium retention percentage was demonstrated in G3 (100 mg Zn-meth) and G4 (20 mg NZnO). Also, dietary zinc in G2 (100 mg ZnSO4) and G3 significantly achieved the highest P retention percentage similar to Attia et al. [9]. On the other hand, compared to chicks fed the basal diet, those fed the ZnS diet (110 mg/kg) showed a significantly higher level of N retention [41]. Our data showed that a 5 mg NZnO/kg diet (G6) significantly had a better Zn retention percentage, followed by nano zinc groups (G4 and G5) and organic zinc methionine groups. The level of zinc used in the broiler feed must correspond with the zinc requirement because a high amount of zinc will not be utilized by the bird’s body, so it is excreted through manure, which initiates environmental pollution [15]. While Mwangi et al. [51] stated that the Zn level in the excreta of chicks given a diet enriched with 40 mg/kg of zinc was higher than that fed a diet containing 8 mg/kg of zinc. The nano zinc sources are expected to decrease the supplemental level of zinc in the diet,consequently, there will be even less zinc released through manure. Compared to the Zn methionine group, the group administered zinc sulfate had a higher level of zinc in their excreta due to a minimal absorption rate [29]. Numerous studies have proven that nano zinc has advanced bioavailability that results in a higher retention percentage compared with the traditional sources, confirmed as high concentrations of Zn in the tibia or liver [85, 89]. Similarly, Kumar et al. [41] found that nano ZnO (100 mg/kg diet) had minimal Zn excretion and elevated Zn percentages in blood, liver, and tibia than ZnSO_4_ at the same level. Hidayat et al. [34] showed that nano zinc oxide at 10, 40, and 70 mg/kg diet significantly achieved more Zn retention. However, the body redistributes the retained zinc to the bone, which acts as a mineral reservoir [55]. It was also found that the nanomaterials can be transferred directly to the target organs [18]. This explanation was confirmed by our current data, which stated that nano zinc oxide in G5 and G6 significantly achieved the maximum zinc content in serum and meat of broilers compared to other treatments.

Our results verified that chicks fed with 10 and 5 mg of Zn/kg diet as NZnO had an elevated villous height (VL) and villus width (WD) and achieved the significantly highest ASA among all experimental groups. However, the significantly lowest crypt depth (CD) was noticed in the zinc oxide group. Also, organic zinc methionine had significantly higher ASA than inorganic zinc sources. It is well known that zinc has an important role in repairing the intestinal epithelium by raising the apoptotic resistance and generation rate, resulting in higher villus height in the intestines of birds [71]. The condition of gut health and inadequate nutrient absorption are closely related to the structure of the intestinal mucosa. Hosseindoust et al. [35] found that weaning piglets fed a high zinc diet had longer villus heights in their duodenums, which increased the surface area available for absorption and, consequently, the piglets'ability to digest nutrients. Moreover, our study exhibited that dietary nano zinc oxide significantly enhances zinc retention and increases the effectiveness of Ca, P, and Zn deposition in blood and tissue meat. It's possible that the increased nutrient retention in response to Zn levels strengthened the development of intestinal architecture, which in turn improved nutrient digestibility [57]. Moreover, research has demonstrated that nano zinc is crucial for altering tight junctions and enhancing the barrier functions of intestinal epithelial cells in rats [45]. Furthermore, Hafez et al. [32] revealed that the length and crypt depth in the duodenum, jejunum, and ileum had risen at a level of 40 and 80 ppm of nano zinc oxide. Moreover, Mocchegiani et al. [48] showed that zinc has a variety of impacts, including promoting the proliferation of intestinal crypt cells, enhancing the cells’ turnover and repair, and supporting the integrity of the intestinal barrier. Also, Ali et al. [3] found that villus height and villus surface area (VSA) in the small intestine were significantly raised by 40 NZnO treatment. In the Jejunum, these parameters were also enhanced in 80 NZnO.

Finally, the improved efficacy at lower doses is likely to be due to the better solubility and interaction of nano ZnO with biological membranes, leading to high absorption and utilization. This allows for reduced supplementation levels without compromising performance and may also minimize environmental zinc excretion. Thus, the ideal diet of 5 mg Zn/kg found in our study corresponds with increasing evidence that highlights the enhanced bioavailability of nano ZnO, allowing for efficient supplementation at reduced concentrations.

## Conclusion

Adding nano zinc oxide at a level of 10 and 5 mg/kg diet to the broilers’ diet as a source of zinc had no adverse impact on body health. It increases carcass yield and improves the growth rate and carcass quality, as proved by a reduction in abdominal fat percentage. Also, it positively impacts breast and thigh meat quality by achieving the highest crude protein and lowest crude fat content. Furthermore, it augments the retention of zinc and improves the efficacy of calcium, phosphorus, and zinc deposition in the serum and meat of birds. Additionally, it is capable of increasing absorption surface area of the intestine, which in turn enhances nutrient digestibility. Therefore, using zinc oxide nanoparticles at a level of 10 and 5 mg/kg diet in broiler nutrition gives a novel advantage compared to inorganic or organic forms.

## Data Availability

Data will be made available on reasonable request.
